# Modulating the Chemical and Sensory Profile of Avgoustiatis Grapes (*Vitis Vinifera* L.) and Wines: The Impact of Irrigation and Post-Harvest Dehydration Under Extreme Mediterranean Thermal Stress

**DOI:** 10.3390/foods15122223

**Published:** 2026-06-20

**Authors:** Despina Lola, Christina Karadimou, Theodoros Gkrimpizis, Dimitrios-Evangelos Miliordos, Kostas Nikolakis, Serafeim Theocharis, Niki Proxenia, Stefanos Koundouras, Yorgos Kotseridis

**Affiliations:** 1 Laboratory of Enology and Alcoholic Drinks, Department of Food Science and Human Nutrition, Agricultural University of Athens, 75 Iera Odos, 11855 Athens, Greece; dim.miliordos@gmail.com (D.-E.M.); nikolakiskostas@gmail.com (K.N.); nprox@aua.gr (N.P.); 2 Laboratory of Viticulture, School of Agriculture, Aristotle University of Thessaloniki, 54124 Thessaloniki, Greece; cckaradi@agro.auth.gr (C.K.); gkrimpiz@agro.auth.gr (T.G.); skoundou@agro.auth.gr (S.K.); 3 Laboratory of Viticulture, Department of Agriculture, International Hellenic University, 57400 Thessaloniki, Greece; stheocharis@ihu.gr

**Keywords:** *Vitis vinifera* L. Avgoustiatis, deficit irrigation, withering, wine quality, polyphenols, aroma compounds, organoleptic characteristics

## Abstract

This study evaluates regulated deficit irrigation (IR) and post-harvest dehydration (DH) as complementary strategies to mitigate extreme thermal stress on the red grape variety Avgoustiatis during the hot 2024 vintage. Analysis of the berries reveals that while IR significantly expanded vine productivity to 2.75 kg/vine compared to 1.32 kg/vine recorded in control vines (CO), it successfully maintained berry weight (240 g). Conversely, DH induced controlled water loss, reducing berry weight to 93 g and concentrating must sugars to 27.3 ^°^Brix, relative to the 23.2 ^°^Brix observed in IR. Crucially, both IR and DH prevented the thermal degradation of total acidity (6.73 g/L and 7.25 g/L respectively) which caused by heat stress in CO samples (6.21 g/L). In the finished wines, both practices increased colour intensity by lowering anthocyanin extractability. However, chemical profiling clearly differentiated the treatments with DH maximized skin tannins (164.7 mg/L), yielding highly structured, astringent wines characterized by plum aromas driven by elevated nerol content (492.91 μg/L). Conversely, IR wines presented a more complex volatile profile, boosting fruity and floral notes. In conclusion, as irrigation becomes increasingly restricted by water scarcity under climate change, post-harvest dehydration offers an effective alternative for producing premium, structurally dense red wines.

## 1. Introduction

The advantageous soil and climatic conditions present in the Greek terroir have significantly contributed to the early advancement of viticulture, with evidence of vine cultivation tracing back to the Neolithic period [1,2]. Over the centuries, a remarkable varietal richness has emerged, with more than 300 indigenous Greek varieties meticulously catalogued in the field of ampelography [3,4]. Despite this extensive genetic diversity, global viticulture exhibits a striking concentration, where just 13 varieties account for thirty-three percent of the globe’s officially recorded vineyard acreage [5]. This reliance on a limited group of international cultivars poses a significant risk in the current era of environmental instability. As a result, native grape varieties are gaining worldwide attention. This vast genetic pool serves as a crucial asset for developing strategies to confront and mitigate the effects of climate change. Indigenous genotypes, having adapted over millennia to the specific microclimates of the Mediterranean, offer unique physiological traits that are essential for sustainable viticulture in a warming world [6,7,8].

In the Mediterranean basin, the anticipated prolongation of summer droughts and rising temperatures, often exceeding 35 °C, is expected to adversely affect grape production and quality by altering the concentration of sugars, acids, and polyphenols [9,10]. For rare indigenous varieties such as Avgoustiatis, a red grape primarily cultivated in small, family-owned plots in the Western Peloponnese and on the island of Zakynthos, managing these extreme heatwaves presents a notable difficulty because of the scarcity of phenological and physiological data [11,12]. The unique vineyard structure of Greece necessitates the adoption of innovative, sustainable techniques that align with each region’s distinctive terroir. To guarantee grape quality under the prevailing climate change, viticultural strategies must focus on the precise modulation of the berry’s chemical matrix through technological interventions that compensate for thermal stress.

A primary scientific strategy currently employed to counteract the intensity of Mediterranean summer droughts is precision irrigation management, specifically Regulated Deficit Irrigation (RDI). Within semi-arid regions characterized by limited water availability, RDI is essential for maximizing water use efficiency by providing a controlled percentage of the calculated crop evapotranspiration (ETc) [13]. This approach provides a technical framework to manipulate berry size and ripening kinetics, ensuring that limited water supplies are utilized to sustain vital physiological functions without inducing excessive vegetative vigour. By precisely tuning the vine’s water status during specific developmental stages, viticulturists can favour the concentration of secondary metabolites, including tannins and anthocyanins, which constitute essential compounds for producing stable and structured red wines of premium quality [14,15,16].

As a complementary strategy to field-based management, post-harvest dehydration (withering) offers an alternative pathway to concentrate and modulate berry components. It is scientifically vital to distinguish between rapid “drying,” which leads to immediate cell death, and slow “withering,” where berry cells remain alive and metabolically active [17]. This post-harvest physiology involves a sequence of complex metabolic events that rearrange the berry’s chemical profile. For instance, dehydration can trigger the biosynthesis of specific compounds, as a broader physiological reaction of the berry water loss [18,19].

For a variety like Avgoustiatis, which is characterized by its high phenolic potential [11], the choice between these two strategies, precision irrigation and post-harvest concentration, is dictated by the specific enological targets of the producer. While irrigation management typically favours aromatic clarity and the retention of natural acidity [20], dehydration protocols deliver structural depth and concentrated plum-driven aromas [21]. Given that many indigenous Greek varieties are cultivated in drought-prone systems, understanding the synergy between these interventions is crucial for preserving their commercial viability and refining the wine’s mouthfeel by balancing the aggressive tannic character that can result from excessive concentration. In this light, the present investigation aims to evaluate the sensory and chemical profile of Avgoustiatis, exploring how these contrasting strategies can be utilized to reposition premium Greek varieties as resilient alternatives to international cultivars. The primary aim of this study is to assess how deficit irrigation and post-harvest drying influence the chemical and organoleptic properties of Avgoustiatis grapes and their resulting wines, providing a robust framework for quality management under extreme Mediterranean thermal stress.

## 2. Materials and Methods

### 2.1. Vineyard and Regional Data

Field experiments were conducted throughout the 2024 vegetative cycle on Zakynthos Island (Ionian Sea, Western Greece), the principal and historic cultivation hub for the native red grapevine cultivar Avgoustiatis (*Vitis vinifera* L.) [12]. The regional climate is categorized as Mediterranean (Köppen Csa), featuring mild, wet winters paired with warm, dry summers. A distinctive element of this coastal mesoclimate is its high annual rainfall, which generally averages between 800 and 1000 mm. While these precipitation events occur mostly during the vine’s dormancy, elevated relative humidity throughout the growing season plays a critical role in shaping grape ripening and phenolic development.

To account for the island’s pedoclimatic diversity, three distinct experimental vineyards were selected, which were also utilized in our parallel study focusing on the defoliation effect of Avgoustiatis grapes and wines: Agios Kirikos (37°47′03.5″ N, 20°49′134 22.5″ E), Katastari (37°49′35.5″ N, 20°45′32.9″ E), and Kalpaki (37°45′45.0″ N, 20°51′42.9″ E) [22]. These trial plots were located at elevations ranging from 30 to 120 m above sea level. Soil composition across the sites was consistently identified as calcareous clay-loam, exhibiting alkaline pH levels (7.5–7.9) and intermediate water retention capabilities—a characteristic highly relevant to the irrigation regimes assessed in this work.

The study utilized mature grapevines (over 12 years old) grafted onto 1103 Paulsen (*V. rupestris* × *V. berlandieri*) rootstocks. These vines were established at a density of roughly 3800 plants per hectare using a 2.4 × 1.2 m spacing grid, trained via vertical shoot positioning (VSP), and spur-pruned to a bilateral Royat cordon.

Beyond the specific irrigation and post-harvest dehydration protocols under investigation, all other viticultural practices remained consistent with standard commercial regional management to ensure that the results are comparable.

### 2.2. Environmental Conditions

The ripening period of 2024 was defined by extreme Mediterranean thermal conditions, as the summer was officially recorded among the hottest in the region’s history [23,24]. During the critical month of July, the vineyard was subjected to three successive heatwaves, during which ambient temperatures frequently exceeded 40 °C. These conditions imposed a severe evaporative demand and significantly influenced the phenological progression of the grapes. Local meteorological data, including temperature and rainfall, were continuously archived by an on-site automated weather station to provide a detailed record of environmental conditions during the trial. A comprehensive summary of these meteorological and climatic parameters is presented in Table S1 of the Supplementary Material.

### 2.3. Vineyard Treatments

A randomized block design was implemented, utilizing a common control to evaluate two distinct strategies for quality modulation under heat stress. The Control (CO) treatment comprised vines grown under the region’s typical rainfed (non-irrigated) conditions. To assess the impact of water relief, a deficit irrigation (IR) treatment was established, wherein supplemental irrigation was initiated at berry set until one week prior to harvest. This regime was designed to mitigate the intensity of the summer drought by providing approximately 50% of the calculated crop evapotranspiration (ETc) based on the Hargreaves and Samani reference model [25]. The irrigation system utilized pressure-compensating emitters located every 50 cm along the lateral pipe to distribute water on either side of each trunk, amounting to a seasonal total of 425 mm during the 2024 growing period.

Simultaneously, a Post-harvest Dehydration (DH) treatment was conducted using fruit sourced from the control vines. Upon reaching technological maturity, intact bunches were manually collected and transferred to a shaded and well-ventilated facility for natural air-drying, following an Amarone-style protocol. The clusters were arranged in single layers within perforated crates to maximize airflow while protecting them from direct sunlight to preserve the integrity of the phenolic and aromatic profile. The dehydration process lasted for a total of 16 days, under controlled room conditions with a mean temperature of 21 ± 2 °C and a relative humidity of 57 ± 5%. The drying progress was tracked via daily weight measurements until the clusters reached a constant mass, indicating that no further moisture loss occurred. At this point, they were immediately processed for vinification.

### 2.4. Experimental Vinification

Micro-scale vinification was conducted in triplicate for each treatment (Control, Deficit Irrigation, and Post-harvest Dehydration) at the experimental winery facilities of the Agricultural University of Athens. The harvest date was scheduled independently for every treatment and replicate to guarantee that all fruits were collected at an equivalent stage of technological maturity. For each replicate, approximately 30 kg of grapes (representing the minimum operational volume calculated for the experimental micro-fermenters, selected due to restricted grape yield caused by adverse weather conditions) were harvested at technological maturity and immediately subjected to mechanical destemming and crushing. The resulting pomace was transferred to stainless-steel fermenters and supplemented with 30 mg/L sulphur dioxide (SO_2_). Following a short pre-fermentative settling period, the musts were inoculated with selected commercial Bravo BV-33 *Saccharomyces cerevisiae* strain (200 mg/L, Renaissance Yeast, Zug, Switzerland), a high-alcohol-tolerant strain to ensure complete conversion of the elevated initial sugar concentrations, even in the dehydrated grape must. To optimize fermentation kinetics, nitrogenous nutrition consisting of diammonium phosphate (DAP) and organic nitrogen (Springferm, Fermentis, Marcq-En-Baroeul, France) was applied in two distinct stages: 24 h post-inoculation and again when once approximately 33% of the initial sugar content had been metabolized.

Alcoholic fermentation (AF) was carried out under a regulated temperature of 23–25 °C. Throughout this phase, phenolic extraction was optimized by manual cap management (punch-downs) executed two to three times per day, with the frequency progressively reduced as the fermentation neared its end. After the residual sugar content fell below 2 g/L, the wine was separated from the pomace using a hydraulic press and inoculated with a commercial *Oenococcus oeni* strain (Viniflora CiNe, CHR Hansen, Hørsholm, Denmark) to induce malolactic fermentation (MLF). Following malic acid depletion (<0.3 g/L), the wines were racked, adjusted to a final SO_2_ concentration of 30 mg/L, and maintained at 5 °C for two months for tartaric and protein stabilization. Lastly, the finished wines were packaged in 750 mL amber glass bottles with a nitrogen (N_2_) gas blanket to avoid oxidation, and aged in a climate-controlled cellar at 15 °C prior to analysis.

### 2.5. Grape Sampling and Physicochemical Analysis

At the stage of technological maturity, representative grape samples were collected from each treatment across the three experimental vineyards. As each vineyard served as a biological replicate, the results are presented as the mean value of the three sites to account for pedoclimatic variability. In particular, 10 clusters were randomly sampled per replicate in accordance with standard viticultural sampling guidelines to record morphological parameters, including bunch dimensions and peduncle length [26,27]. From the remaining bulk, 200 berries were randomly collected from each replicate, providing a standard, statistically representative and sufficient sample size for technological and phenolic characterization. To prevent enzymatic degradation and metabolic shifts in berries selected for phenolic evaluation, they were transported to the laboratory on dry ice and stored at −20 °C until analysis.

Representative berry samples from each replicate were pressed to obtain the must, which was subsequently analysed for total soluble solids (^°^Brix), pH, and titratable acidity (TA). All measurements were performed in accordance with the official standard methods of the OIV [28].

### 2.6. Conventional Wine Analysis

The experimental wines underwent characterization by assessing core oenological parameters, including alcoholic strength by volume (vol%), residual sugars concentration, pH, titratable acidity (TA), and volatile acidity (VA). To guarantee a thorough evaluation of the cultivar’s enological potential and its reaction to the various irrigation and withering treatments, all analyses were carried out in triplicate in accordance with standard OIV protocols [28].

### 2.7. Chemical and Phenolic Analysis

For the determination of phenolic and chromatic properties, skins and seeds were manually separated from 50-berry replicates, freeze-dried, and ground into a homogeneous fine powder. Phenolics were then isolated from 0.3 g of this pulverized tissue utilizing a two-stage sequential extraction protocol. The matrix was initially treated with 2.5 mL of an acetone/water mixture (80:20, *v*/*v*) for 3 h, and subsequently subjected to a second extraction phase using 2.5 mL of methanol/water (60:40, *v*/*v*) for a further 2.5 h. The pooled supernatants were concentrated under reduced pressure at 30 °C and subsequently freeze-dried to obtain the crude phenolic extracts [29]. While the majority of the assays utilized lyophilized materials, total anthocyanins and phenolic extractability indices were measured directly using intact berries. For both grape and wine samples, the Total Polyphenolic Index (TPI) was determined spectrophotometrically via absorbance at 280 nm [30]. Concurrently, total phenolic content was evaluated with the Folin–Ciocalteu colorimetric assay at 750 nm, with values quantified against a standard curve and reported as gallic acid equivalents (GAE) [31]. Total and extractable anthocyanins were evaluated following the Iland and Glories protocols, respectively, to determine the pigments’ potential and extractability [32,33]. To quantify the tannin profile, the Methylcellulose Precipitation (MCP) assay (recorded at 280 nm) was employed for both grape and wine samples. For a more detailed characterization of wine tannins, the Bovine Serum Albumin (BSA) protein precipitation assay (monitored at 510 nm) was additionally performed as a complementary method [34,35]. Finally, wine chromatic characteristics were evaluated spectrophotometrically to determine Colour Intensity and Hue, reflecting the overall depth and tonality of the wine [30]. All spectrophotometric analyses were conducted in triplicate using a Hitachi U-2000 spectrophotometer (Jasco, Victoria, BC, Canada) to ensure analytical precision and reproducibility.

### 2.8. Volatile Composition Analysis

Characterization of the Avgoustiatis wine aroma compounds was achieved using Gas Chromatography-Mass Spectrometry (GC-MS) via a liquid-liquid extraction technique modified from Goulioti et al. [36]. For every experimental group, a 40 mL wine sample was fortified with three internal standards—3-octanol, ethyl heptanoate, and heptanoic acid—to yield an individual final concentration of 10 mg/L. Volatile metabolites were isolated in triplicate through the addition of 5 mL of dichloromethane, accompanied by 15 min of continuous magnetic agitation. The organic fraction was separated via centrifugation (4000 rpm, 10 min, 4 °C), and this extraction loop was duplicated to optimize compound recovery. The organic extracts were then dehydrated over anhydrous sodium sulfate and reduced to 500 μL using a mild stream of nitrogen gas.

Chromatographic separation was performed utilizing a GC-MS apparatus consisting of a Perkin Elmer Clarus 590 GC system integrated with a Clarus SQ8S MS detector and an HTA S.R.L. autosampler. Volatile components were resolved on a polar capillary Agilent DB-WAX column (50 m × 0.25 mm i.d., 0.25 μm film thickness). The injection port temperature was maintained at 250 °C, and helium was utilized as the carrier gas at a fixed flow rate (1.0 mL/min). The oven temperature program was initiated at 40 °C for 2 min, ramped to 240 °C at 5 °C/min, and held at the final temperature for 20 min. Mass spectra were acquired across a scan range of 40–400 m/z. Volatile compounds were identified by comparing their mass spectra with the National Institute of Standards and Technology (NIST) library and by verifying retention times against authentic commercial standards (Figure S1). Quantification was performed using external seven-point calibration curves established for each specific compound.

### 2.9. Sensory Analysis

The sensory evaluation was conducted at the Laboratory of Enology and Alcoholic Drinks of the Agricultural University of Athens, involving a panel of 12 highly trained assessors with a balanced gender representation (ages 25–57). The sessions took place three months after bottling in individual booths under controlled environmental conditions (20–22 °C), typically between 11:00 a.m. and 1:00 p.m. to ensure maximum concentration. Prior to the formal trials, the panellists underwent a comprehensive three-week training period comprising three sessions, during which they were familiarized with aroma and taste standards and calibrated on the intensity of various descriptors to ensure panel homogeneity [37]. All assessors successfully completed the training sessions and participated in the evaluation session in accordance with the following experimental protocol approved by the Research Ethics Commission of the Agricultural University of Athens.

Wine samples (30 mL) were presented at room temperature in ISO 3591 standard glasses, which were capped with glass lids and identified via randomized three-digit codes. A monadic presentation order following a Latin Square design was utilized to mitigate positional bias and sensory fatigue [37]. Judges evaluated the intensity of designated attributes using a 10-point scale (1 to 10), spannining visual (colour intensity and hue), olfactory (aroma intensity, red fruits, plum, violet, vanilla, pepper, and vegetative), and palatal traits (acidity, body, balance, and astringency). To avoidsensory carryover, a mandatory 5 min inter-sample interval was enforced, during which assessors rinsed their palates with water. The sensorial test and data collection was managed digitally using the Compusense Cloud software, Version 24.0.55 (Compusense, Guelph, ON, Canada) No assessor exhibited anomalous behaviour or insufficient performance warranting exclusion from the study prior to data treatment. Therefore, all sensory evaluations were retained for statistical analysis, and the final sensory profile for each wine was calculated as the mean of the evaluation sessions, providing a robust quantitative assessment of the samples’ oenological quality.

### 2.10. Statistical Analysis

All determinations were performed in triplicate, and results are expressed as mean values ± standard deviation (SD). To evaluate the effects of the viticultural treatments (control, deficit irrigation and post-harvest dehydration), the data were subjected to one-way analysis of variance (ANOVA). Significant differences between treatments were identified using Tukey’s HSD post-hoc test at the 95% confidence level (*p* < 0.05). For the sensory evaluation data, the assumptions for parametric analysis were assessed (normality using the Shapiro–Wilk test, and homogeneity of variance using Levene’s test), and as they were not met, non-parametric analysis was employed to identify significant differences among treatments. The Kruskal–Wallis test was followed by Dunn’s multiple-comparison procedure, and the resulting sensory profiles were visualized using spider plots and Hierarchical Cluster Analysis (HCA) with Euclidean distance and Ward’s agglomerative clustering method, implemented in XLSTAT. Furthermore, a Heatmap combined with HCA was generated to illustrate the relationships between the different viticultural and post-harvest treatments and the resulting wine volatile profiles. Pearson’s correlation coefficients were determined between the concentrations of volatile compounds (individual compounds and total volatile-compound groups) and the sensory evaluation scores of aroma descriptors. All statistical processing and graphical representations were carried out using IBM SPSS Statistics (version 26) and XLSTAT (Addinsoft, Paris, France, 2017).

## 3. Results and Discussion

### 3.1. Meteorological Conditions

The extreme heat conditions observed in 2024 are corroborated by the meteorological data presented in Supplementary Material (Table S1). Notably, the absolute maximum temperature peaked at 42.2 °C in July. Furthermore, a prolonged period of severe thermal stress was recorded from May through September, with absolute maximum temperatures consistently exceeding 34.0 °C. These extreme thermal values, coupled with negligible summer precipitation (averaging < 8 mm/month from June to August), confirm the intensity and duration of the heat during the study period.

### 3.2. Yield and Morphological Parameters of Grapes

The viticultural practices applied significantly influenced the yield and morphological characteristics of the Avgoustiatis variety (Table 1). Irrigation (IR) led to a significant increase in productivity, with a yield of 2.75 kg/vine compared to 1.32 kg/vine in control (CO). This increase, representing approximately a 108% rise in production, aligns with established findings regarding the stabilizing effect of irrigation on grapevine productivity in Mediterranean semi-arid environments, where water resources represent the principal limiting constraint [38]. While the IR treatment showed a trend toward higher berry weight (240 g per 50 units) than the CO treatment (210 g), this variation did not reach statistical significance. This suggests that for Avgoustiatis, yield expansion under irrigation may be more closely linked to increased fruit set or cluster numbers rather than solely berry expansion, a phenomenon observed in other red cultivars under regulated deficit irrigation (RI) [39,40]. In contrast, post-harvest dehydration (DH) significantly reduced berry weight to 93 g. This drastic reduction is a signature of off-vine withering, where weight loss is driven primarily by water evaporation from the berry, though it is also accompanied by carbon loss due to ongoing cellular respiration during the early stages of the process [41].

Regarding cluster morphology, IR resulted in significantly shorter bunches (15.3 cm) compared to CO (18.6 cm), while bunch width and peduncle length remained statistically similar across treatments. The non-significant difference in midday leaf water potential (Ψ_leaf_) in July (−1.30 MPa for CO and −0.95 MPa for IR) indicates that while the CO vines were under higher water pressure, they remained within the range of moderate-to-severe stress typical for rain-fed Mediterranean vineyards [38].

### 3.3. Must Composition and Grape Phenolic Potential

The composition of Avgoustiatis must, and the phenolic potential of the grapes, were significantly modulated by the applied viticultural and post-harvest practices. The post-harvest dehydration (DH) treatment resulted in the highest sugar concentration (27.3 ^°^Brix), which was significantly higher than the irrigation (IR) treatment (23.2 ^°^Brix), while the control (CO) remained intermediate (24.5 ^°^Brix). This increase in soluble solids during DH is a well-documented effect of water evaporation from berries, which leads to the concentration of primary metabolites [42]. Conversely, the lower sugar levels in IR must reflect a dilution effect associated with higher vine productivity and berry weight expansion under maintained hydration. Regarding acidity, both IR (6.73 g/L) and DH (7.25 g/L) treatments resulted in significantly higher total acidity (TA) than the CO treatment (6.21 g/L), whereas notable variations were also detected in must pH. The elevated TA in IR must suggest that irrigation mitigated the thermal degradation of malic acid by reducing canopy temperatures, while the increase in the DH treatment is attributed to the concentration of organic acids alongside sugars (Table 2) [43].

The phenolic potential of the grapes and the ease of their extraction were significantly modulated by the applied viticultural and post-harvest interventions, as detailed in Table 3. Total phenolics reached their significantly highest concentration in the dehydration (DH) treatment (2320 mg GAE/L), while the control (CO) showed the lowest values (1750 mg GAE/L). The irrigation (IR) treatment (2150 mg GAE/L) followed an intermediate trend. The elevated concentration in DH grapes is primarily attributed to the significant water loss and subsequent concentration of solutes. However, literature suggests that post-harvest withering is not merely a physical concentration process; the berries remain metabolically active, which can lead to shifts in the phenolic profile as cells respond to water-loss stress [44,45].

A critical finding in this study was the significant impact on anthocyanin extractability (%). A progressive and significant decrease was observed from the control (34.2%) to the IR (22.1%) and finally to the DH treatment (16.6%). Given that the AE% index functions inversely, this decrease indicates a significant enhancement in the ease of anthocyanin extraction for the IR and DH treatments. The superior extractability in the DH grapes is likely due to the degradation of skin cell wall polysaccharides during the post-harvest period, which renders the anthocyanins more accessible during vinification [44]. Similarly, the irrigation treatment likely facilitated a more balanced physiological ripening compared to the control. By mitigating seasonal water deficits, irrigation supports the natural enzymatic degradation of the skin cell wall matrix, which in turn enhances the release of anthocyanins during vinification without the structural impediments often found in berries from non-irrigated vines [46].

Complementing the extractability trends, the absolute total anthocyanin content per berry was significantly influenced by the treatments, with the DH group reaching the highest level (1.68 mg/berry). While the concentration effect of water loss plays a role, the increase in anthocyanin content per berry compared to the IR samples (1.26 mg/berry) suggests that metabolic activity during the dehydration phase may have triggered late-stage biosynthesis or a specific stress response [47]. When viewed alongside the AE% results, it is evident that the DH treatment not only results in a higher quantity of pigments per unit of fruit but also ensures they are in a state that is more readily available for diffusion into the liquid phase during winemaking.

Regarding the tannin profile, the post-harvest dehydration also exerted a profound effect, particularly on skin tannins, which surged to 164.7 g/L in the DH treatment—a significant increase over both the CO (113.6 g/L) and IR (105.8 g/L) samples. This enrichment in skin tannins is primarily attributed to the concentration effect resulting from substantial water loss during the dehydration process, which increases the density of phenolic compounds per unit of volume [48]. Interestingly, seed tannins were also significantly higher in both the IR (180.4 g/L) and DH (180.6 g/L) treatments compared to the control (156.3 g/L). For the IR group, this may be related to differences in berry size and seed maturation under non-stressed conditions, whereas for the DH group, it reflects a highly concentrated phenolic load.

Ultimately, these findings highlight a strategic distinction between the two interventions: while post-harvest dehydration provides the maximum concentration and technological extractability for the final product, irrigation emerges as a vital viticultural tool for preserving the absolute phenolic content per berry and maintaining vine equilibrium under the extreme thermal conditions of the Mediterranean climate. Collectively, these results indicate that combining appropriate field-level water management with controlled post-harvest drying can produce a raw material with superior phenolic potential.

### 3.4. Physicochemical Parameters and Colour Evolution of Produced Wines

The primary oenological parameters of the wines (Table 4) closely mirror the chemical profiles observed in the raw material during ripening. All fermentations reached dryness (>0.03 g/L residual sugar) with a consistent alcoholic strength of 13.3% vol, indicating that the sugar levels at harvest were effectively converted regardless of the viticultural treatment. A significant convergence was observed in total acidity; the post-harvest dehydration (DH) wines exhibited the highest values (5.29 g/L), directly reflecting the concentration of organic acids (tartaric and malic) previously detected in the dehydrated berries. This confirms that the dehydration process is primarily a concentration of solutes, as these acids are not metabolized during the withering phase [48]. Despite the high solute density, DH wines maintained the lowest volatile acidity (0.47 g/L), indicating high microbial stability. While pH values were relatively high across all samples (3.98–4.18), this trend is consistent with the extreme thermal stress and high potassium levels characteristic of the 2024 Mediterranean season [23,49].

**Figure 1:**
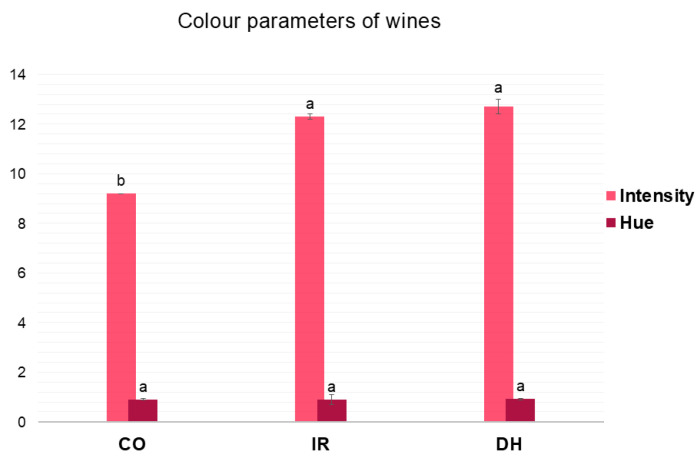
The effect of applied viticultural practices on wine colour characteristics (CO: control, IR: Deficit Irrigation, DH: Post-harvest dehydration). Values are means ± SD of triplicate fermentations. The mean values followed by different lowercase letters indicate significant differences according to the Tukey HSD test (*p* < 0.05).

The chromatic results (Figure 1) strongly align with the anthocyanin extractability indices measured in the grapes. Both irrigation (IR) and dehydration (DH) significantly enhanced wine colour intensity relative to the control, consistent with the superior extractability findings. For the IR treatment, the high colour intensity in the wine supports the grape-level hypothesis that mitigating water deficits promotes more balanced physiological ripening and a more permeable skin structure, thereby optimizing pigment release. In the DH wines, the peak intensity converges with the increased absolute anthocyanin content (1.68 mg/berry). Hue values remained stable at 0.9, suggesting that neither treatment induced premature oxidative browning during the vinification of the Avgoustiatis variety. Similar to our findings, several authors have demonstrated that deficit-irrigated and dehydrated grapes produce higher colour in rose and red wines [50,51,52].

### 3.5. Phenolic Content and Tannin Fractions of Produced Wines

**Figure 2:**
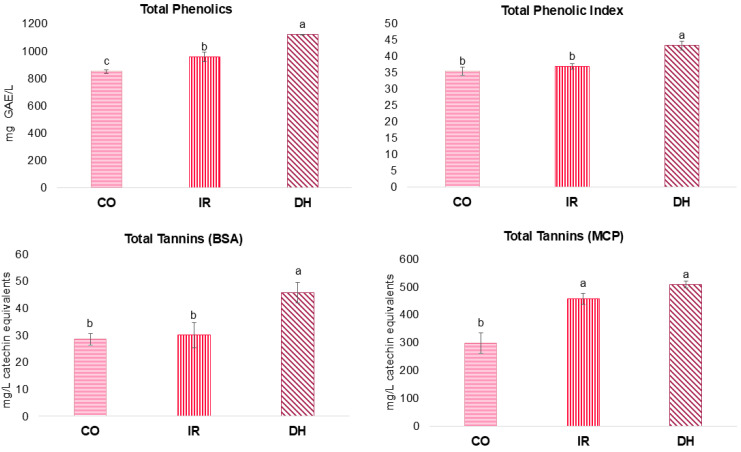
The effect of applied viticultural practices (CO: control, IR: Deficit Irrigation, DH: Post-harvest dehydration) on wine phenolic characteristics (TPI: total phenolic index, TP: total phenolics as mg GAE/L) and tannic content (Tannins measured with the MCP and BSA methods, expressed as mg/L catechin equivalents). Values are means± SD of triplicate fermentations. The mean values followed by different lowercase letters indicate significant differences according to the Tukey HSD test (*p* < 0.05).

The phenolic potential of the wines was significantly modulated by the applied practices, as the wines’ phenolic profile (Figure 2) shows a robust correlation with the berries’ initial potential. The Total Phenolic Index (TPI) and total phenolics (mg GAE/L) reached their maximum in the DH treatment, directly aligning with the high concentrations of secondary metabolites found in the dehydrated skins. This convergence confirms that the “concentration effect” is the dominant factor in the phenolic enrichment of DH wines, as the absolute quantity of tannins and pigments per berry is preserved and concentrated within a smaller volume of must. Supporting our results, previous research shows that postharvest water loss triggers extensive metabolic shifts within grape berries, which subsequently alter the chemical composition of the resulting wine, particularly by increasing its phenolic concentration [44,51,53].

Regarding the tannin structure, the wine data confirms the distinctions observed in the grape seeds and skins. Total tannins (MCP method) were significantly higher in both DH and IR wines, which is consistent with the higher phenolic load of these treatments compared to the control. However, a specific convergence emerged in the BSA-reactive fraction, which is linked to astringency. Only the DH wines exhibited a significant increase in aggressive tannins, mirroring the high skin tannin concentration (164.7 g/L) in the dehydrated grapes. In contrast, the IR treatment increased total phenolics and MCP-tannin levels while maintaining BSA-tannin levels similar to the control. This provides a vital oenological insight: while dehydration maximizes the total phenolic potential, irrigation appears to facilitate a high-quality extraction of “riper” tannins, providing structure without the excessive bitterness often associated with highly concentrated, stressed berries.

### 3.6. Volatile Composition of Avgoustiatis Wines

The GC–MS analysis of wines enabled the identification and quantification of both varietal and fermentation-derived compounds, including ethyl esters and acetates, higher alcohols, acids, and terpenes. Table 5 presents the mean concentrations of volatile compounds identified in the studied wines and the results of the one-way ANOVA.

Esters are mainly formed enzymatically by yeast during alcoholic fermentation, and they are responsible for fruity aromas. Their concentration is influenced by the composition of the must and the fermentation conditions [54]. In this research, the effect of the treatment applied to the samples was not significant for most esters, with concentrations in this important qualitative group remaining similar across the wines. Only ethyl octanoate showed a significant reduction in wines produced from vine drying and deficit irrigation compared to the control. Some studies have reported that the formation of total esters is directly proportional to the amount of amino acids, and our findings could be explained more clearly if the nitrogen or amino acid content of the must had been identified [55,56,57]. It is worth noting that opposite trends have been observed in ester contents regarding the applied treatments. Previous evidence on post-harvest dehydration consistently shows an increase in ester compounds, especially ethyl esters, in the resulting wines [58,59]. This enrichment results from active metabolic reprogramming during dehydration rather than from passive concentration due to water loss. Importantly, grape variety and dehydration conditions—such as temperature, air humidity, duration, dehydration rate, and sugar levels—further influence the ester profile [60]. Notably, extreme dehydration, which leads to very high sugar levels, may paradoxically reduce some ester classes during fermentation, even though the dehydration process itself promotes their precursors in the grape [60,61]. Irrigation timing, intensity, and cultivar influence the ester content in wines as well. Our findings are similar to those of a study showing that deficit irrigation had no effect on ester concentrations [62]. However, previous studies show that less water-fed vines and deficit irrigation enhance branched-chain amino acid availability, which in turn feeds ester biosynthesis during fermentation. On the other hand, full irrigation tends to dilute ester concentration and reduce aromatic intensity, making it not recommended at any phenological stage [63,64].

Higher alcohols were the most abundant group of volatile compounds across all wines in the study. For secondary products of yeast metabolism, such as isoamyl alcohol, 2-methyl-1-propanol, 2-phenylethanol, and 3-methylthiolpropanol, which are derived mainly via the Ehrlich pathway [65], concentrations were similar across wines. Additionally, given that higher alcohols in concentrations above 300 mg/L may negatively affect overall wine aroma [30,66], none of the samples in this study contained compounds exceeding this concentration. Therefore, it can be affirmed that they contribute positive nuances to the aroma of Avgoustiatis wines. By contrast, C6 alcohols are generated through the oxidation of polyunsaturated fatty acids found in grapes (e.g., oleic, linoleic, linolenic) via the lipoxygenase (LOX) pathway when the berries are crushed [65]. Levels of these compounds were significantly higher in the control wines, whereas *cis*-3-hexenol was absent from wines obtained after irrigation and postharvest dehydration in the vineyard. Findings in the literature regarding the content of C6 alcohols after postharvest drying are inconsistent, suggesting that grape exposure temperature is a key factor influencing the biological activity of lipoxygenase (LOX) enzymes and, consequently, the final levels of these compounds in grapes and wines. A study on Malvasia grapes reported an increase in LOX activity accompanied by a parallel rise in C6 compounds during postharvest drying, representing an initial metabolic response that peaked at 11.7% weight loss [17]. However, at more advanced stages of dehydration, these compounds may become undetectable, as observed in Pedro Ximénez grapes and in the wines from our study, indicating reduced LOX activity in the berry during off-vine drying [67]. Regarding irrigation treatment, previous studies have demonstrated a decrease in C6 volatile concentrations in Verdejo and Tempranillo wines obtained from irrigated vines [68,69].

Volatile acids are a class of organic acids in wine, primarily derived from the grapes themselves and from microbial metabolism during winemaking. Moderate levels of volatile acids not only increase the complexity and interest of wine but also help maintain good stability and shelf life [61]. Shinohara [70] reported that concentrations of 4–10 mg/L of C6–C10 fatty acids provided a mild and pleasant aroma for wine, while levels above 20 mg/L had a negative impact on sensory attributes. In our study, both viticultural strategies significantly increased acid concentrations compared with the control, but all wines remained below this level. More specifically, volatile acids showed the highest concentrations under deficit irrigation treatment, confirming previous studies indicating that moderate irrigation increases volatile fatty acid contents [71], especially when water availability is moderate during a dry season [68]. Regarding post-dehydration treatment, an increase in fatty acids was observed, consistent with the study by Piombino et al. [72]. In general, our findings underscore the importance of water-loss kinetics and cultivar identity as key modulators of volatile acid content, as shown in previous studies on off-vine drying of Nebbiolo, Aleatico, and Marsecan varieties [61,72].

As with the terpenoid group, the three terpenoid compounds (nerol, geraniol, β-damascenone) identified and quantified in all samples were more abundant in wines from the viticultural treatments, though the difference was not statistically significant for β-damascenone. Terpenes are grape-derived compounds that contribute substantially to the wine’s olfactory profile, even in neutral varieties, because of their “flowery” and “fruity” attributes [73]. Wines from postharvest dehydration showed a significant increase in nerol content, supporting previous work indicating the accumulation of terpenes in dehydrated berries due to the up-regulation of related biosynthetic genes and, consequently, in wines [72,74,75,76]. Yet the same process simultaneously causes the loss of the most abundant free monoterpenes due to their transformation during alcoholic fermentation and aging. For example, Slaghenaufi et al. [59] reported a significant decrease in free linalool and geraniol, with linalool oxides produced through the oxidation of linalool, a biochemical process typically stimulated by dehydration, and a possible contribution of geraniol to free citronellol levels due to the availability of yeasts to reduce geraniol content. This phenomenon may explain the proportionally smaller increase in geraniol compared with the increase in nerol in the DH wines of our study. As far as the response of terpenes to vine-level deficit irrigation is considerably more consistent across the literature, it clearly shows a trend toward enhancement. Several authors suggest that light to moderate water stress increases the concentration of monoterpenes such as limonene, linalool, α-terpineol, geranyl acetone, citronellol, nerol, and geraniol, and that there is a positive relationship between the concentration of C13-norisoprenoids, such as β-damascenone, β-ionone, and 1,1,6-trimethyl-1,2-dihydronaphthalene, and moderate to severe water deficit in several red wine varieties [69,77,78,79,80,81]. This is likely because moderate water deficit also triggers terpenoid synthase expression at the transcriptomic level, and the intensity of this effect varies with the timing of water stress and the variety [82,83].

**Figure 3:**
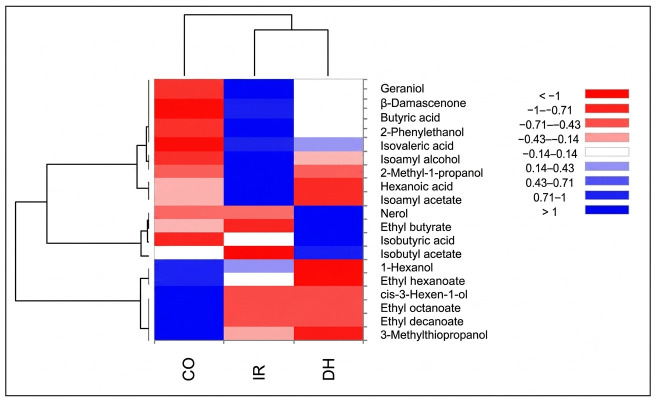
Heatmap analysis based on the concentrations of volatile compounds identified in Avgoustiatis wines obtained from the different viticultural treatments (CO: Control, IR: Deficit Irrigation, DH: Post-harvest dehydration).

Heatmap analysis was used to further examine differences in volatile compounds among Avgoustiatis wines produced under different viticultural treatments. Figure 3 shows a double clustering pattern: wines cluster by treatment, and volatile compounds cluster by their concentrations in the analysed samples. More specifically, a clear separation was observed between the control wine and the IR and DH wines, indicating substantial differences in their volatile profiles. Furthermore, distinct volatile compositions were observed between the IR and DH wines.

A similar clustering pattern was observed for the aromatic compounds, which were grouped into three main clusters according to their concentration profiles. The first cluster contained 1-hexanol, ethyl hexanoate, *cis*-3-hexen-1-ol, ethyl octanoate, ethyl decanoate, and 3-methylthiopropanol, compounds that generally presented higher levels in the control samples and relatively stable trends in wines from the other two treatments. The second cluster included nerol, ethyl butyrate, isobutyric acid, and isobutyl acetate, which showed the highest concentrations in wines from the dehydration treatment, while exhibiting moderate or low levels in the wines from the control and irrigation treatments. The third cluster comprised geraniol, β-damascenone, butyric acid, 2-phenylethanol, isovaleric acid, isoamyl alcohol, 2-methyl-1-propanol, and isoamyl acetate, compounds that were present at the highest concentrations in wines produced from water-deficient vines.

### 3.7. Sensory Evaluation of Wines

**Figure 4:**
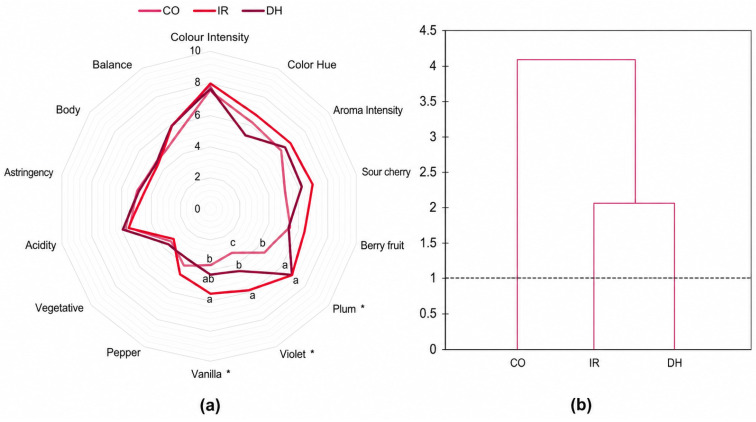
(**a**) Spider plot of the means of the sensory characteristics of the Avgoustiatis wines produced from the different viticultural treatments. Significant differences between samples (CO, IR, DH) per average mean intensity of descriptors determined by the Kruskal-Wallis test followed by the Dunn post hoc test (*p* < 0.05) and indicated by the different lowercase letters and the asterisk (*). (**b**) Dendrogram obtained by HCA using a dissimilarity matrix applied to sensory data resulting from the descriptive analysis evaluation of the Avgoustiatis wines produced from the different viticultural treatments. CO: control, IR: Deficit Irrigation, DH: Post-harvest dehydration.

All experimental Avgoustiatis wines were assessed by a panel of sensory experts to highlight the variations in volatile compound levels among wines made with the different viticultural treatments mentioned above. Sensory evaluation of the wines was conducted using quantitative descriptive analysis. The results (Figure 4a) showed significant differences in aroma characteristics among Avgoustiatis wines produced using distinct viticultural strategies, whereas no significant variations were detected regarding colour properties (intensity, hue) or mouthfeel attributes (balance, body, astringency, acidity). Wines from both viticultural treatments, especially those from water deficit (IR), scored higher in aroma intensity, sour cherry, and berry fruit attributes than the control samples. Notably, these scores were significantly higher for plum, vanilla, and violet aroma traits, which may reflect the differences in the wines’ volatile substances. Indeed, deficit irrigation and dehydration treatments produced many compounds that enhanced the wines’ characteristics and aromatic complexity, including terpenes and acids. Pearson’s correlation test highlighted some links between the aroma descriptors and the compound concentrations found in wine samples (Table S2). More specifically, the content of total acids and nerol showed a positive correlation with plum (0.84 and 0.62, respectively), while the contents of β-damascenone and geraniol were positively correlated with the attributes of plum (0.85 and 0.84, respectively), vanilla (0.97 and 0.98, respectively), and violet (0.98 and 0.99, respectively) (Table S2). Additionally, the similar ester profiles among the studied wines likely led to nearly equal perceptions of sour cherry and berry fruit aromas [84,85], and the lower perception in the control wines could be due to their higher abundance of C_6_ alcohols, which can negatively affect fruity perceptions at high concentrations. Supporting this finding, a negative correlation was observed between *cis*-3-hexen-1-ol and plum aroma descriptor (−0.95, Table S2). Furthermore, several higher alcohols and volatile acids, including 2-phenylethanol, isoamyl alcohol, butyric acid, and isovaleric acid, were positively correlated with aroma intensity and with descriptors such as sour cherry, vanilla, and violet (Table S2). Their relatively higher abundance in wines from deficit-irrigated vineyards (Figure 3, Table 5), although not always statistically significant, is consistent with enhanced perception of these sensory attributes compared with wines produced from postharvest-dehydrated grapes. These relationships further support the association between the wines’ volatile composition and their sensory profiles. In agreement with our study, similar results showing more fruity nuances and an intense aroma in wines produced from deficit-irrigated grapes have been reported in the literature [81,86,87]. With respect to dehydration, prior studies have demonstrated that volatile profiles are modified both qualitatively and quantitatively due to the synthesis of novel odour-active compounds that contribute jammy, dark fruit, plum, floral, and caramelized aromatic notes [88,89,90].

Overall, the observed differences and trends in sensory characteristics were further highlighted by hierarchical cluster analysis (HCA), which grouped the wines into the same three clusters identified in the heatmap analysis (Figure 3), based on odour similarities. Indeed, the HCA (Figure 4b) confirmed the presence of distinct sensory groups associated with the chemical composition of wines derived from different viticultural practices.

## 4. Conclusions

The present study provides a thorough characterization ofdeficit irrigation and post-harvest dehydration as complementary quality-management strategies for the indigenous Greek grape variety Avgoustiatis under the extreme thermal stress conditions of the 2024 Mediterranean growing season. Both approaches effectively modulated grape and wine composition, although through distinct mechanisms and toward different oenological objectives. Deficit irrigation preserved vine productivity and must acidity under severe heat stress while enhancing anthocyanin extractability and promoting balanced skin ripening. In contrast, post-harvest dehydration, applied using an Amarone-style withering protocol, resulted in the highest concentration of primary and secondary metabolites, including soluble solids, phenolics, tannins, and anthocyanins. These compositional differences were reflected in the wines, with dehydrated grapes producing wines with the highest phenolic and tannin content. Sensory differences were mainly associated with aroma. Wines produced under vine deficit irrigation showed increased levels of higher alcohols and terpenoids, including geraniol, contributing to fruity and floral notes, whereas post-harvest dehydration increased nerol content and promoted plum and dark-fruit aromas. Overall, irrigation appears better suited to producing wines with freshness, acid retention, and tannic elegance, while dehydration favours more structured wines with concentrated phenolic and aromatic profiles. Considering the increasing limitation of water resources under climate change, post-harvest dehydration may represent a technically effective alternative to irrigation for premium wine production. Importantly, Avgoustiatis demonstrated considerable phenolic potential and physiological adaptability under both strategies, supporting its suitability as a resilient Mediterranean cultivar.

Finally, our results are based on a single growing season and three experimental vineyards, which aligns with the vineyard constraints on Zakynthos Island and the warm Mediterranean conditions. However, a larger number of sites and vintages would improve the generalizability of our findings. Furthermore, the post-harvest dehydration treatment was applied exclusively to grapes from control vines, so potential interactions with irrigation management cannot be assessed. Therefore, future studies should focus on extending these findings to the combined application of these techniques across different vintages, terroirs, and additional vineyard sites. Similarly, profiling individual phenolic compounds would provide additional insight into the specific phenolic pathways modulated by these viticultural practices and would be a promising direction for future research.

## Figures and Tables

**Table 1:** The effect of the applied viticultural practices on grape morphological parameters. Data represent mean ± SD (*n* = 3). The mean values followed by different lowercase letters in the same row are significantly different (*p* < 0.05, Tukey HSD test).

Samples ^a^	Weight of 50 Grapes (g)	Bunch Length (cm)	Bunch Width (cm)	Peduncle Length (cm)	Leaf Water Potential (MPa, July)	Yield (kg)/Vine
CO	210 ± 8 a	18.6 ± 0.2 a	8.91 ± 0.14 a	1.18 ± 0.07 a	−1.30 ± 0.22 a	1.32 ± 0.29 b
IR	240 ± 12 a	15.3 ± 0.1 b	8.54 ± 0.55 a	1.34 ± 0.05 a	−0.95 ± 0.17 a	2.75 ± 0.41 a
DH	93 ± 4 b	-	-	-	-	-

^a^ CO: control, IR: Deficit Irrigation, DH: Post-harvest dehydration.

**Table 2:** Avgoustiatis grape must composition obtained by the untreated control samples (CO) and the applied viticultural treatments: Deficit irrigation (IR), Post-harvest dehydration (DH). Data represent mean ± SD (*n* = 3). Different lowercase letters in a single row denote statistically significant differences between means (*p* < 0.05, Tukey HSD test).

Samples ^a^	Brix	pH	Total Acidity (Tartaric Acid g/L)
CO	24.5 ± 0.2 ab	3.65 ± 0.0 a	6.21 ± 0.11 b
IR	23.2 ± 0.1 b	3.55 ± 0.1 a	6.73 ± 0.14 ab
DH	27.3 ± 0.4 a	3.18 ± 0.2 b	7.25 ± 0.22 a

^a^ CO: control, IR: Deficit Irrigation, DH: Post-harvest dehydration.

**Table 3:** The effect of the applied viticultural practices on phenolic composition of Avgoustiatis grapes. Data are expressed as mean ± SD (*n* = 3). Means within the same row bearing distinct lowercase letters indicate significant differences (*p* < 0.05, Tukey HSD test).

Samples ^a^	Total Phenolics (mg GAE/L)	Extractability (%)	Total Anthocyanins (mg/Berry)	Total Tannins MCP (g/L)	
				skins	seeds
CO	1750 ± 21 b	34.2 ± 2.1 a	1.33 ± 0.24 ab	113.6 ± 9.7 b	156.3 ± 15.2 b
IR	2150 ± 18 ab	22.1 ± 1.7 b	1.26 ± 0.18 ab	105.8 ± 10.0 b	180.4 ± 12.5 a
DH	2320 ± 29 a	16.6 ± 1.8 c	1.68 ± 0.10 a	164.7 ± 4.2 a	180.6 ± 7.8 a

^a^ CO: control, IR: Deficit Irrigation, DH: Post-harvest dehydration.

**Table 4:** Impact of the applied viticultural treatments on the enological parameters of the resulting wines. Data are expressed as mean ± SD (*n* = 3) from triplicate fermentations. Means within the same row bearing distinct lowercase letters indicate significant differences (*p* < 0.05, Tukey HSD test).

Samples ^a^	Residual Sugar (g/L)	Alcohol (Vol%)	Total Acidity (Tartaric Acid g/L)	pH	Volatile Acidity (Acetic Acid g/L)
CO	0.01 ± 0.0 a	13.3 ± 0.0 a	4.31 ± 0.14 b	4.18 ± 0.07 a	0.59 ± 0.02 a
IR	0.03 ± 0.01 a	13.3 ± 0.1 a	4.39 ± 0.14 b	3.98 ± 0.08 a	0.57 ± 0.02 a
DH	0.02 ± 0.01 a	13.3 ± 0.1 a	5.29 ± 0.19 a	4.07 ± 0.15 a	0.47 ± 0.05 b

^a^ CO: control, IR: Deficit Irrigation, DH: Post-harvest dehydration.

**Table 5:** Average concentration (± SD) of volatile compounds in Avgoustiatis wines produced under various viticultural treatments (*n* = 9; derived from triplicate fermentations and triplicate analyses). The mean values followed by different lowercase letters in the same row are significantly different (*p* < 0.05, Tukey HSD test).

Volatile Compounds	CO ^a^	IR ^a^	DH ^a^
Esters (mg/L)			
Isobutyl acetate	0.12 ± 0.01 a	0.09 ± 0.00 a	0.15 ± 0.04 a
Isoamyl acetate	0.40 ± 0.00 ab	0.41 ± 0.01 a	0.40 ± 0.00 b
Ethyl butyrate	0.86 ± 0.02 a	0.82 ± 0.04 a	1.00 ± 0.13 a
Ethyl hexanoate	0.23 ± 0.00 a	0.23 ± 0.00 a	0.22 ± 0.01 a
Ethyl octanoate	0.63 ± 0.00 a	0.23 ± 0.00 b	0.22 ± 0.00 b
Ethyl decanoate	0.14 ± 0.03 a	0.11 ± 0.00 a	0.11 ± 0.00 a
Total esters	2.19 ± 0.25 a	1.83 ± 0.09 a	2.11 ± 0.09 a
Alcohols (mg/L)			
2-Methyl-1-propanol	33.63 ± 0.44 a	46.99 ± 6.72 a	34.78 ± 3.48 a
Isoamyl alcohol	103.92 ± 8.35 a	116.96 ± 15.28 a	107.64 ± 9.00 a
2-Phenylethanol	9.26 ± 1.16 a	12.11 ± 3.89 a	10.40 ± 1.96 a
1-Hexanol	1.45 ± 0.10 a	1.39 ± 0.10 ab	1.20 ± 0.05 b
cis-3-Hexen-1-ol	0.15 ± 0.03	n.d.	n.d.
3-Methylthiopropanol	0.42 ± 0.07 a	0.37 ± 0.05 a	0.35 ± 0.01 a
Total alcohols	140.38 ± 16.90 a	165.64 ± 38.48 a	154.38 ± 13.72 a
Acids (mg/L)			
Isobutyric acid	3.18 ± 0.22 b	3.84 ± 0.33 ab	4.85 ± 0.42 a
Butyric acid	1.23 ± 0.08 c	1.90 ± 0.03 a	1.57 ± 0.12 b
Isovaleric acid	1.71 ± 0.13 b	2.49 ± 0.33 a	2.21 ± 0.14 ab
Hexanoic acid	1.62 ± 0.06 ab	2.08 ± 0.25 a	1.47 ± 0.11 b
Total acids	6.64 ± 2.07 a	8.26 ± 3.81 a	10.10 ± 0.58 a
Terpenoids (μg/L)			
Nerol	308.26 ± 10.75 b	327.16 ± 41.76 b	492.91 ± 28.30 a
β-Damascenone	0.55 ± 0.13 a	0.74 ± 0.14 a	0.64 ± 0.10 a
Geraniol	7.75 ± 0.41 b	10.48 ± 1.02 a	9.02 ± 0.04 ab
Total terpenoids	314.07 ± 13.16 a	335.02 ± 40.19 a	501.93 ± 28.34 a

^a^ CO: Control, IR: Deficit Irrigation, DH: Post-harvest dehydration.

## Data Availability

The original contributions presented in this study are included in the article/Supplementary Material. Further inquiries can be directed to the corresponding authors.

## Supplementary Materials

The following supporting information can be downloaded at: https://www.mdpi.com/article/10.3390/foods15122223/s1, Table S1. Mean climatic data recorded at Zakynthos meteorological station (2024); Table S2. Pearson’s correlations between concentrations of aroma compounds in wines and the intensity scores of descriptors obtained by sensory evaluation; Figure S1. Representative GC–MS total ion chromatogram (TIC) of Avgoustiatis wine samples.

## Abbreviations

The following abbreviations are used in this manuscript:

TPI	Total Polyphenolic Index
CO	Control
ΙR	Irrigation treatment
DH	Post dehydration treatment
DAP	Diammonium Phosphate
AF	Alcoholic Fermentation
MLF	Malolactic Fermentation
TA	Titratable Acidity
VA	Volatile Acidity
GAE	Gallic Acid Equivalents
BSA	Bovine Serum Albumin
MCP	Methylcellulose Precipitation
CI	Colour Intensity
H	Hue
GC–MS	Gas chromatography−Mass spectrometry
ISO	International Organization for Standardization
ANOVA	One-way Analysis of Variance
SD	Standard Deviation
HCA	Hierarchical Cluster Analysis

## References

[1] Pagnoux C., Bouby L., Ivorra S., Petit C., Valamoti S.-M., Pastor T., Picq S., Terral J.-F. (2015). Inferring the Agrobiodiversity of *Vitis vinifera* L. (Grapevine) in Ancient Greece by Comparative Shape Analysis of Archaeological and Modern Seeds. Veg. Hist. Archaeobot..

[2] Avramidou E., Masaoutis I., Pitsoli T., Kapazoglou A., Pikraki M., Trantas E., Nikolantonakis M., Doulis A. (2023). Analysis of Wine-Producing *Vitis vinifera* L. Biotypes, Autochthonous to Crete (Greece), Employing Ampelographic and Microsatellite Markers. Life.

[3] Stavrakas D.E. (2015). Ampelography.

[4] Merkouropoulos G. (2023). Greek Grapevine Varieties. Names in the Greek Bibliography.

[5] OIV State of the World Vine and Wine Sector. https://www.oiv.int/sites/default/files/documents/OIV-State_of_the_World_Vine-and-Wine-Sector-in-2024.pdf.

[6] Wolkovich E.M., García de Cortázar-Atauri I., Morales-Castilla I., Nicholas K.A., Lacombe T. (2018). From Pinot to Xinomavro in the World’s Future Wine-Growing Regions. Nat. Clim. Change.

[7] Morales-Castilla I., García de Cortázar-Atauri I., Cook B.I., Lacombe T., Parker A., van Leeuwen C., Nicholas K.A., Wolkovich E.M. (2020). Diversity Buffers Winegrowing Regions from Climate Change Losses. Proc. Natl. Acad. Sci. USA.

[8] Duchêne E., Huard F., Dumas V., Schneider C., Merdinoglu D. (2010). The Challenge of Adapting Grapevine Varieties to Climate Change. Clim. Res..

[9] Hannah L., Roehrdanz P.R., Ikegami M., Shepard A.V., Shaw M.R., Tabor G., Zhi L., Marquet P.A., Hijmans R.J. (2013). Climate Change, Wine, and Conservation. Proc. Natl. Acad. Sci. USA.

[10] van Leeuwen C., Sgubin G., Bois B., Ollat N., Swingedouw D., Zito S., Gambetta G.A. (2024). Climate Change Impacts and Adaptations of Wine Production. Nat. Rev. Earth Environ..

[11] Papakonstantinou L.D., Sotiropoulos S.S., Taskos D.G., Paschalidis D.C., Chamurliev G.O. (2021). Ampelographic Presentation of Some Indigenous Grape Varieties of Greece. Magarach Vinograd. Vinodel..

[12] Eriotou E., Kopsahelis N., Lappa I., Alimpoumpa D., Diamanti V., Koulougliotis D. (2020). Identification of Indigenous Yeast Strains from Spontaneous Vinification of Grapes from the Red Variety *Avgoustiatis zakynthou* (Ionian Islands, Greece) and Antioxidant Activity of the Produced Wine. J. Food Chem. Nanotechnol..

[13] Alatzas A., Theocharis S., Miliordos D.-E., Leontaridou K., Kanellis A.K., Kotseridis Y., Hatzopoulos P., Koundouras S. (2021). The Effect of Water Deficit on Two Greek *Vitis vinifera* L. Cultivars: Physiology, Grape Composition and Gene Expression during Berry Development. Plants.

[14] Alatzas A., Theocharis S., Miliordos D.-E., Kotseridis Y., Koundouras S., Hatzopoulos P. (2023). Leaf Removal and Deficit Irrigation Have Diverse Outcomes on Composition and Gene Expression during Berry Development of *Vitis vinifera* L. Cultivar Xinomavro. OENO One.

[15] Koundouras S., Marinos V., Gkoulioti A., Kotseridis Y., van Leeuwen C. (2006). Influence of Vineyard Location and Vine Water Status on Fruit Maturation of Nonirrigated Cv. Agiorgitiko (*Vitis vinifera* L.). Effects on Wine Phenolic and Aroma Components. J. Agric. Food Chem..

[16] Savoi S., Wong D.C.J., Arapitsas P., Miculan M., Bucchetti B., Peterlunger E., Fait A., Mattivi F., Castellarin S.D. (2016). Transcriptome and Metabolite Profiling Reveals That Prolonged Drought Modulates the Phenylpropanoid and Terpenoid Pathway in White Grapes (*Vitis vinifera* L.). BMC Plant Biol..

[17] Costantini V., Bellincontro A., De Santis D., Botondi R., Mencarelli F. (2006). Metabolic Changes of Malvasia Grapes for Wine Production during Postharvest Drying. J. Agric. Food Chem..

[18] Panceri C.P., De Gois J.S., Borges D.L.G., Bordignon-Luiz M.T. (2015). Effect of Grape Dehydration under Controlled Conditions on Chemical Composition and Sensory Characteristics of Cabernet Sauvignon and Merlot Wines. LWT—Food Sci. Technol..

[19] Scalzini G., Vernhet A., Carillo S., Roi S., Véran F., Jørgensen B., Hansen J., Giacosa S., Segade S.R., Paissoni M.A. (2024). Cell Wall Polysaccharides, Phenolic Extractability and Mechanical Properties of Aleatico Winegrapes Dehydrated under Sun or in Controlled Conditions. Food Hydrocoll..

[20] Roby G., Harbertson J.F., Adams D.A., Matthews M.A. (2004). Berry Size and Vine Water Deficits as Factors in Winegrape Composition: Anthocyanins and Tannins. Aust. J. Grape Wine Res..

[21] Bellincontro A., De Santis D., Botondi R., Villa I., Mencarelli F. (2004). Different Postharvest Dehydration Rates Affect Quality Characteristics and Volatile Compounds of Malvasia, Trebbiano and Sangiovese Grapes for Wine Production. J. Sci. Food Agric..

[22] Gkrimpizis T., Lola D., Karadimou C., Theocharis S., Chatzidimitriou E., Kotseridis Y., Koundouras S. (2026). Enhancing Oenological Quality of *Vitis vinifera* L. Avgoustiatis: The Effect of Early Leaf Removal on Grape and Wine Composition. Gastronomy.

[23] Ionita M., Vaideanu P., Nichita D., Nagavciuc V. (2025). Breaking Records under Clear Skies: The Impact of Sunshine Duration and Atmospheric Dynamics on the 2024 Eastern European Extreme Summer Temperatures. npj Nat. Hazards.

[24] Androulidakis Y., Kolovoyiannis V., Makris C., Krestenitis Y. (2024). Evidence of 2024 Summer as the Warmest During the Last Four Decades in the Aegean, Ionian, and Cretan Seas. J. Mar. Sci. Eng..

[25] Hargreaves G.H., Samani Z.A. (1982). Estimating Potential Evapotranspiration. J. Irrig. Drain. Eng..

[26] Amerine M.A., Roessler E.B. (1958). Field Testing of Grape Maturity. Hilgardia.

[27] Lucarini M., Durazzo A., Kiefer J., Santini A., Lombardi-Boccia G., Souto E.B., Romani A., Lampe A., Ferrari Nicoli S., Gabrielli P. (2020). Grape Seeds: Chromatographic Profile of Fatty Acids and Phenolic Compounds and Qualitative Analysis by FTIR-ATR Spectroscopy. Foods.

[28] OIV (2021). Compendium of International Methods of Wine and Must Analysis: Volume 1.

[29] Lorrain B., Chira K., Teissedre P.-L. (2011). Phenolic Composition of Merlot and Cabernet-Sauvignon Grapes from Bordeaux Vineyard for the 2009-Vintage: Comparison to 2006, 2007 and 2008 Vintages. Food Chem..

[30] Ribéreau-Gayon P., Glories Y., Maujean A., Dubourdieu D. (2006). Handbook of Enology the Chemistry of Wine Stabilization and Treatments.

[31] Arnous A., Makris D.P., Kefalas P. (2001). Effect of Principal Polyphenolic Components in Relation to Antioxidant Characteristics of Aged Red Wines. J. Agric. Food Chem..

[32] Iland P.G., Cynkar W., Francis I.L., Williams P.J., Coombe B.G. (1996). Optimisation of Methods for the Determination of Total and Red-Free Glycosyl Glucose in Black Grape Berries of *Vitis vinifera*. Aust. J. Grape Wine Res..

[33] Glories Y. (1984). La Couleur Des Vins Rouges. Lre Partie: Les Équilibres Des Anthocyanes et Des Tanins. OENO One.

[34] Harbertson J.F., Picciotto E.A., Adams D.O. (2003). Measurement of Polymeric Pigments in Grape Berry Extract Sand Wines Using a Protein Precipitation Assay Combined with Bisulfite Bleaching. Am. J. Enol. Vitic..

[35] Sarneckis C.J., Dambergs R.G., Jones P., Mercurio M., Herderich M.J., Smith P.A. (2006). Quantification of Condensed Tannins by Precipitation with Methyl Cellulose: Development and Validation of an Optimised Tool for Grape and Wine Analysis. Aust. J. Grape Wine Res..

[36] Goulioti E., Jeffery D.W., Kanapitsas A., Lola D., Papadopoulos G., Bauer A., Kotseridis Y. (2023). Chemical and Sensory Characterization of Xinomavro Red Wine Using Grapes from Protected Designations of Northern Greece. Molecules.

[37] Lola D., Miliordos D.E., Goulioti E., Kontoudakis N., Myrtsi E.D., Haroutounian S.A., Kotseridis Y. (2023). Assessment of the Volatile and Non-Volatile Profile of Savatiano PGI Wines as Affected by Various Terroirs in Attica, Greece. Food Res. Int..

[38] Chacón-Vozmediano J.L., Martínez-Gascueña J., García-Romero E., Gómez-Alonso S., García-Navarro F.J., Jiménez-Ballesta R. (2021). Effects of Water Stress on the Phenolic Compounds of ‘Merlot’ Grapes in a Semi-Arid Mediterranean Climate. Horticulturae.

[39] Munitz S., Netzer Y., Schwartz A. (2017). Sustained and Regulated Deficit Irrigation of Field-Grown Merlot Grapevines. Aust. J. Grape Wine Res..

[40] Valdés M.E., Moreno D., Gamero E., Uriarte D., Del Henar Prieto M., Manzano R., Picón J., Intrigliolo D.S. (2009). Effects of Cluster Thinning and Irrigation Amount on Water Relations, Growth, Yield and Fruit and Wine Composition of Tempranillo Grapes in Extemadura (Spain). OENO One.

[41] Bellincontro A., Mencarelli F. (2022). Postharvest Physiology of Wine Grape Dehydration. Managing Wine Quality.

[42] Tonutti P., Bonghi C. (2013). Biochemistry and Physiology of Dehydrating Berries. Sweet, Reinforced and Fortified Wines.

[43] Ozkan K., Karadag A., Sagdic O., Ozcan F.S., Ozer H. (2023). The Effects of Different Drying Methods on the Sugar, Organic Acid, Volatile Composition, and Textural Properties of Black ‘Isabel’ Grape. J. Food Meas. Charact..

[44] Sanmartin C., Modesti M., Venturi F., Brizzolara S., Mencarelli F., Bellincontro A. (2021). Postharvest Water Loss of Wine Grape: When, What and Why. Metabolites.

[45] Pott D.M., Vallarino J.G., Osorio S. (2020). Metabolite Changes during Postharvest Storage: Effects on Fruit Quality Traits. Metabolites.

[46] Rouxinol M.I., Martins M.R., Barroso J.M., Rato A.E. (2023). Wine Grapes Ripening: A Review on Climate Effect and Analytical Approach to Increase Wine Quality. Appl. Biosci..

[47] Guillaumie S., Fouquet R., Kappel C., Camps C., Terrier N., Moncomble D., Dunlevy J.D., Davies C., Boss P.K., Delrot S. (2011). Transcriptional Analysis of Late Ripening Stages of Grapevine Berry. BMC Plant Biol..

[48] Panceri C.P., Gomes T.M., De Gois J.S., Borges D.L.G., Bordignon-Luiz M.T. (2013). Effect of Dehydration Process on Mineral Content, Phenolic Compounds and Antioxidant Activity of Cabernet Sauvignon and Merlot Grapes. Food Res. Int..

[49] Petoumenou D.G., Liava V. (2025). Sustainable Foliar Applications to Improve Grapevine Responses to Drought, High Temperatures, and Salinity: Impacts on Physiology, Yields, and Berry Quality. Plants.

[50] Chalmers Y.M., Downey M.O., Krstic M.P., Loveys B.R., Dry P.R. (2010). Influence of Sustained Deficit Irrigation on Colour Parameters of Cabernet Sauvignon and Shiraz Microscale Wine Fermentations. Aust. J. Grape Wine Res..

[51] Bai Y., Zhao P., Du Y., Lin J., Han F. (2023). Effect of Postharvest Grape Dehydration on the Phenolic Composition of ‘Marselan’ Rose Wine during Aging. J. Food Compos. Anal..

[52] Urcan D.E., Giacosa S., Torchio F., Río Segade S., Raimondi S., Bertolino M., Gerbi V., Pop N., Rolle L. (2017). ‘Fortified’ Wines Volatile Composition: Effect of Different Postharvest Dehydration Conditions of Wine Grapes Cv. Malvasia Moscata (*Vitis vinifera* L.). Food Chem..

[53] Ossola C., Giacosa S., Torchio F., Río Segade S., Caudana A., Cagnasso E., Gerbi V., Rolle L. (2017). Comparison of Fortified, Sfursat, and Passito Wines Produced from Fresh and Dehydrated Grapes of Aromatic Black Cv. Moscato Nero (*Vitis vinifera* L.). Food Res. Int..

[54] Ferreira V., López R., Cacho J.F. (2000). Quantitative Determination of the Odorants of Young Red Wines from Different Grape Varieties. J. Sci. Food Agric..

[55] Garde-Cerdán T., Ancín-Azpilicueta C. (2008). Effect of the Addition of Different Quantities of Amino Acids to Nitrogen-Deficient Must on the Formation of Esters, Alcohols, and Acids during Wine Alcoholic Fermentation. LWT—Food Sci. Technol..

[56] Rapp A., Versini G. (1995). Influence of Nitrogen Compounds in Grapes on Aroma Compounds of Wines. Dev. Food Sci..

[57] Lola D., Kalloniati C., Dimopoulou M., Kanapitsas A., Papadopoulos G., Dorignac E., Flemetakis E., Kotseridis Y. (2023). Impact of Assimilable Nitrogen Supplementation on Saccharomyces Cerevisiae Metabolic Response and Aromatic Profile of Moschofilero Wine. J. Agric. Food Chem..

[58] López de Lerma N., Moreno J., Peinado R.A. (2014). Determination of the Optimum Sun-Drying Time for *Vitis vinifera* L. Cv. Tempranillo Grapes by E-Nose Analysis and Characterization of Their Volatile Composition. Food Bioproc. Technol..

[59] Slaghenaufi D., Boscaini A., Prandi A., Dal Cin A., Zandonà V., Luzzini G., Ugliano M. (2020). Influence of Different Modalities of Grape Withering on Volatile Compounds of Young and Aged Corvina Wines. Molecules.

[60] Li R., Tong W., Liu Y., Ge Q., Xu X., Yu K., Shi W., Mu H., Yan G., Duan C. (2026). Sugar Stress Attenuates Fruity Aroma in Sweet Wine by Suppressing Ethyl Ester Biosynthesis: Insights from Integrated Sensory, Metabolome, and Transcriptomic Analyses. Food Chem. X.

[61] Xi C., Zhang J., Zhang F., Liu D., Cheng W., Gao F., Wang P. (2024). Effect of Postharvest Grape Dehydration on Chemical Composition, Antioxidant Activity and Sensory Characeteristics of Marselan Wines. Food Chem. X.

[62] Qian M.C., Fang Y., Shellie K. (2009). Volatile Composition of Merlot Wine from Different Vine Water Status. J. Agric. Food Chem..

[63] Baeza P., Junquera P., Peiro E., Ramón Lissarrague J., Uriarte D., Vilanova M. (2019). Effects of Vine Water Status on Yield Components, Vegetative Response and Must and Wine Composition. Advances in Grape and Wine Biotechnology.

[64] Ju Y., Xu G., Yue X., Zhao X., Tu T., Zhang J., Fang Y. (2018). Effects of Regulated Deficit Irrigation on Amino Acid Profiles and Their Derived Volatile Compounds in Cabernet Sauvignon (*Vitis vinifera* L.) Grapes and Wines. Molecules.

[65] Robinson A.L., Boss P.K., Solomon P.S., Trengove R.D., Heymann H., Ebeler S.E. (2014). Origins of Grape and Wine Aroma. Part 1. Chemical Components and Viticultural Impacts. Am. J. Enol. Vitic..

[66] Sánchez-Palomo E., García-Carpintero E.G., Viñas M.A.G. (2015). Aroma Fingerprint Characterisation of La Mancha Red Wines. S. Afr. J. Enol. Vitic..

[67] Franco M., Peinado R.A., Medina M., Moreno J. (2004). Off-Vine Grape Drying Effect on Volatile Compounds and Aromatic Series in Must from Pedro Ximénez Grape Variety. J. Agric. Food Chem..

[68] Vilanova M., Rodríguez-Nogales J.M., Vila-Crespo J., Yuste J. (2019). Influence of Water Regime on Yield Components, Must Composition and Wine Volatile Compounds of *Vitis vinifera* Cv. Verdejo. Aust. J. Grape Wine Res..

[69] Talaverano I., Valdés E., Moreno D., Gamero E., Mancha L., Vilanova M. (2017). The Combined Effect of Water Status and Crop Level on Tempranillo Wine Volatiles. J. Sci. Food Agric..

[70] Shinohara T. (1985). Gas Chromatographic Analysis of Volatile Fatty Acids in Wines. Agric. Biol. Chem..

[71] Talaverano I., Ubeda C., Cáceres-Mella A., Valdés M.E., Pastenes C., Peña-Neira Á. (2018). Water Stress and Ripeness Effects on the Volatile Composition of Cabernet Sauvignon Wines. J. Sci. Food Agric..

[72] Piombino P., Pittari E., Genovese A., Bellincontro A., Moio L. (2025). Postharvest Dehydration of Red Grapes: Impact of Temperature and Water-loss Conditions on Free and Glycosylated Volatile Metabolites of Exocarp and Epicarp of Nebbiolo and Aleatico Varieties. J. Sci. Food Agric..

[73] Tufariello M., Capone S., Siciliano P. (2012). Volatile Components of Negroamaro Red Wines Produced in Apulian Salento Area. Food Chem..

[74] Moreno J.J., Cerpa-Calderón F., Cohen S.D., Fang Y., Qian M., Kennedy J.A. (2008). Effect of Postharvest Dehydration on the Composition of Pinot Noir Grapes (*Vitis vinifera* L.) and Wine. Food Chem..

[75] Shmuleviz R., Amato A., Commisso M., D’Incà E., Luzzini G., Ugliano M., Fasoli M., Zenoni S., Tornielli G.B. (2023). Temperature Affects Organic Acid, Terpene and Stilbene Metabolisms in Wine Grapes during Postharvest Dehydration. Front. Plant Sci..

[76] Zenoni S., Fasoli M., Guzzo F., Dal Santo S., Amato A., Anesi A., Commisso M., Herderich M., Ceoldo S., Avesani L. (2016). Disclosing the Molecular Basis of the Postharvest Life of Berry in Different Grapevine Genotypes. Plant Physiol..

[77] Brillante L., Martínez-Lüscher J., Kurtural S.K. (2018). Applied Water and Mechanical Canopy Management Affect Berry and Wine Phenolic and Aroma Composition of Grapevine (*Vitis vinifera* L., Cv. Syrah) in Central California. Sci. Hortic..

[78] Bindon K.A., Dry P.R., Loveys B.R. (2007). Influence of Plant Water Status on the Production of C 13 -Norisoprenoid Precursors in *Vitis vinifera* L. Cv. Cabernet Sauvignon Grape Berries. J. Agric. Food Chem..

[79] Koundouras S., Hatzidimitriou E., Karamolegkou M., Dimopoulou E., Kallithraka S., Tsialtas J.T., Zioziou E., Nikolaou N., Kotseridis Y. (2009). Irrigation and Rootstock Effects on the Phenolic Concentration and Aroma Potential of *Vitis vinifera* L. Cv. Cabernet Sauvignon Grapes. J. Agric. Food Chem..

[80] Song J., Shellie K.C., Wang H., Qian M.C. (2012). Influence of Deficit Irrigation and Kaolin Particle Film on Grape Composition and Volatile Compounds in Merlot Grape (*Vitis vinifera* L.). Food Chem..

[81] Savoi S., Herrera J.C., Carlin S., Lotti C., Bucchetti B., Peterlunger E., Castellarin S.D., Mattivi F. (2020). From Grape Berries to Wines: Drought Impacts on Key Secondary Metabolites. OENO One.

[82] Deluc L.G., Decendit A., Papastamoulis Y., Mérillon J.-M., Cushman J.C., Cramer G.R. (2011). Water Deficit Increases Stilbene Metabolism in Cabernet Sauvignon Berries. J. Agric. Food Chem..

[83] Palai G., Caruso G., Gucci R., D’Onofrio C. (2023). Water Deficit before Veraison Is Crucial in Regulating Berry VOCs Concentration in Sangiovese Grapevines. Front. Plant Sci..

[84] Pineau B., Barbe J.-C., Van Leeuwen C., Dubourdieu D. (2009). Examples of Perceptive Interactions Involved in Specific “Red-“ and “Black-Berry” Aromas in Red Wines. J. Agric. Food Chem..

[85] Lytra G., Franc C., Cameleyre M., Barbe J.-C. (2017). Study of Substituted Ester Formation in Red Wine by the Development of a New Method for Quantitative Determination and Enantiomeric Separation of Their Corresponding Acids. J. Agric. Food Chem..

[86] Casassa L., Keller M., Harbertson J. (2015). Regulated Deficit Irrigation Alters Anthocyanins, Tannins and Sensory Properties of Cabernet Sauvignon Grapes and Wines. Molecules.

[87] Cáceres-Mella A., Ribalta-Pizarro C., Villalobos-González L., Cuneo I.F., Pastenes C. (2018). Controlled Water Deficit Modifies the Phenolic Composition and Sensory Properties in Cabernet Sauvignon Wines. Sci. Hortic..

[88] Tomasi D., Lonardi A., Boscaro D., Nardi T., Marangon C.M., De Rosso M., Flamini R., Lovat L., Mian G. (2021). Effects of Traditional and Modern Post-Harvest Withering Processes on the Composition of the Vitis v. Corvina Grape and the Sensory Profile of Amarone Wines. Molecules.

[89] Noguerol-Pato R., González-Álvarez M., González-Barreiro C., Cancho-Grande B., Simal-Gándara J. (2013). Evolution of the Aromatic Profile in Garnacha Tintorera Grapes during Raisining and Comparison with That of the Naturally Sweet Wine Obtained. Food Chem..

[90] Chou H.-C., Šuklje K., Antalick G., Schmidtke L.M., Blackman J.W. (2018). Late-Season Shiraz Berry Dehydration That Alters Composition and Sensory Traits of Wine. J. Agric. Food Chem..

