# Supplementary material for: Modulating the Chemical and Sensory Profile of Avgoustiatis Grapes (Vitis Vinifera L.) and Wines: The Impact of Irrigation and Post-Harvest Dehydration Under Extreme Mediterranean Thermal Stress
Source: Foods. 2026 Jun 20;15(12):2223. doi: 10.3390/foods15122223 (PMC13297933; doi:10.3390/foods15122223)
Supplement: Supplementary file 1 [file foods-15-02223-s001.zip › foods-4348762-supplementary.pdf]

Article

Modulating the Chemical and Sensory Profile of Avgoustiatis Grapes (*Vitis Vinifera* L.) and Wines: The Impact of Irrigation and Post-Harvest Dehydration Under Extreme Mediterranean Thermal Stress

Despina Lola [1,*], Christina Karadimou [2], Theodoros Gkrimpizis [2], Dimitrios-Evangelos Miliordos [1], Kostas Nikolakis [1], Serafeim Theocharis [3], Niki Proxenia [1], Stefanos Koundouras [2] and Yorgos Kotseridis [1,*]

[1] Laboratory of Enology and Alcoholic Drinks, Department of Food Science and Human Nutrition, Agricultural University of Athens, 75 Iera Odos, 11855 Athens, Greece; dim.miliordos@gmail.com (D.-E.M.); nikolakiskostas@gmail.com (K.N.); nprox@aua.gr (N.P.)
[2] Laboratory of Viticulture, School of Agriculture, Aristotle University of Thessaloniki, 54124 Thessaloniki, Greece; cckaradi@agro.auth.gr (C.K.); gkrimpiz@agro.auth.gr (T.G.); skoundou@agro.auth.gr (S.K.)
[3] Laboratory of Viticulture, Department of Agriculture, International Hellenic University, 57400 Thessaloniki, Greece; stheocharis@ihu.gr
* Correspondence: despinalola@gmail.com (D.L.); ykotseridis@aua.gr (Y.K.)

Table of Contents **Page**

Table S1. Mean climatic data recorded at Zakynthos meteorological station (2024)...2
Table S2. Pearson's correlations between concentrations of aroma compounds in wines and the intensity scores of descriptors obtained by sensory evaluation 2
Figure S1. Representative GC–MS total ion chromatogram (TIC) of Avgoustiatis wine samples 3

Table S1. Mean climatic data recorded at Zakynthos meteorological station (2024)[a].

Parameter	Jan.	Feb.	Mar.	Apr.	May	Jun.	Jul.	Aug.	Sep.	Oct.	Nov.	Dec.	YEAR
Atmospheric pressure (hPa)	1016.9	1015.1	1014.8	1013.5	1014.0	1013.5	1012.6	1012.8	1015.5	1017.0	1017.1	1016.5	1014.9
Mean temperature (°C)	11.3	11.5	12.9	15.5	19.8	24.1	26.7	26.6	23.8	19.6	15.8	12.8	18.4
Mean maximum temperature (°C)	14.4	14.5	16.1	18.9	23.4	27.8	30.7	30.6	27.6	23.0	19.0	15.8	21.8
Mean minimum temperature (°C)	8.1	8.2	9.2	11.1	14.4	18.2	20.4	20.9	18.8	15.7	12.5	9.6	13.9
Absolute maximum temperature (°C)	20.2	21.4	24.2	25.6	34.2	35.8	42.2	38.4	36.8	30.4	26.6	22.2	–
Absolute minimum temperature (°C)	-2.6	-2.0	0.0	2.6	5.0	8.4	12.0	13.4	10.8	5.2	2.8	0.2	–
Mean relative humidity (%)	74	73	73	72	68	63	59	61	67	72	76	75	69
Mean precipitation (mm)	150	113	90	51	17	7	5	9	25	147	159	170	943
Mean number of rainy days (> 1 mm)	13	11	8	6	3	1	1	1	3	8	11	13	79
Mean wind speed (m/s)	2.2	2.4	2.2	1.9	1.8	1.8	2.0	1.9	1.8	2.1	2.0	2.2	2.0

[a]Source: Hellenic National Meteorological Service. Values represent long-term monthly means. (–) indicates data not available.

Table S2. Pearson's correlations between concentrations of aroma compounds in wines and the intensity scores of descriptors obtained by sensory evaluation.

Aroma compounds	Aroma attributes	Pearson coefficients	p-value
2-phenylethanol	Aroma intensity	+ 0.99	0.015
isovaleric acid	Sour cherry	+ 0.82	0.056
total alcohols		+ 0.91	0.013
geraniol	Plum	+ 0.84	0.045
β-damascenone		+ 0.85	0.039
nerol		+ 0.62	0.046
total acids		+ 0.84	0.043
cis-3-hexen-1-ol		- 0.95	0.004
geraniol	Vanilla	+ 0.97	0.047
β-damascenone		+ 0.91	0.012
isoamyl alcohol		+ 0.96	0.034
2-phenylethanol		+ 0.98	0.049
geraniol	Violet	+ 0.99	0.022
β-damascenone		+ 0.98	0.022
butyric acid		+ 0.96	0.015
total alcohols		+ 0.92	0.042

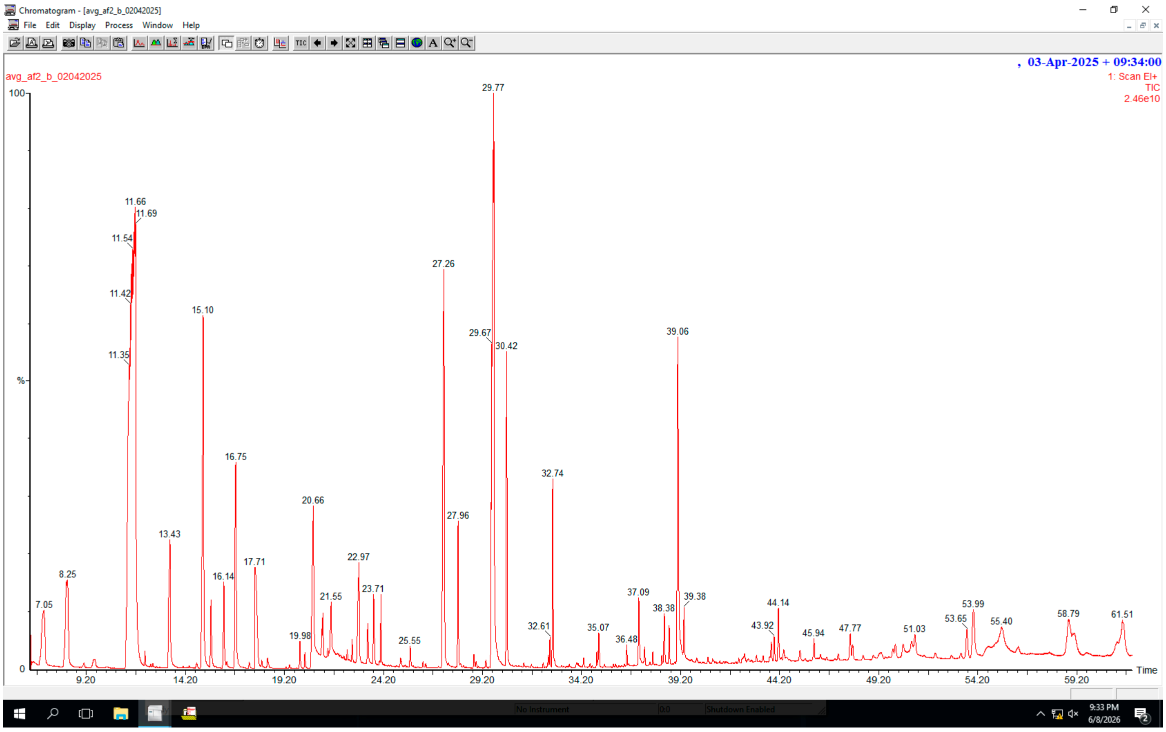

Figure S1. Representative GC–MS total ion chromatogram (TIC) of Avgoustiatis wine samples.
